# Innovation Strategies of the Spanish Agri-Food Sector in Response to the Black Swan COVID-19 Pandemic

**DOI:** 10.3390/foods9121821

**Published:** 2020-12-08

**Authors:** Margarita Brugarolas, Laura Martínez-Carrasco, Adrián Rabadán, Rodolfo Bernabéu

**Affiliations:** 1 Escuela Politécnica Superior de Orihuela (EPSO), Universidad Miguel Hernández, Avenida de la Universidad de Elche s/n, 03202 Elche, Spain; lmartinez@umh.es; 2 Escuela Técnica Superior de Ingenieros Agrónomos y de Montes (ETSIAM), Universidad de Castilla-La Mancha, Campus Universitario s/n, 02071 Albacete, Spain; adrian.rabadan@uclm.es (A.R.); Rodolfo.Bernabeu@uclm.es (R.B.)

**Keywords:** agri-food marketing, consumer behaviour, economic and social crisis, health, innovation

## Abstract

Health, financial, and social crises cause variations in the buying behaviour of food consumers as well as in the value they assign to food attributes and the place of purchase, leading to consumers with profiles that are more susceptible to these changes than others. Thus, it was observed that 61.4% of consumers modified their buying behaviour at the onset of the COVID-19 pandemic, with those who modified it the most being the people who stockpiled the most food and went panic buying more often. This has made it possible to establish the profile of different significant consumer segments, and as a response, food production/distribution companies can implement different innovative strategies aimed at decreasing the impact of stockpiling and, therefore, the shortage of food. The possible strategies that companies can put into effect are creating a stock of non-perishable foods, increasing production capabilities in a sustainable way and, especially in light of the results obtained, boost the online sale and distribution of foods, with the goal of decreasing the amount of people in shops (which decreases the spreading of the pandemic and favours health) and preventing consumers from observing possible circumstantial shortages that would only encourage stockpiling and panic buying, even among consumers who have not changed their buying behaviour.

## 1. Introduction

Black swan is a term coined by investor Nassim Nicholas Taleb in 2007 and it has been used to designate unpredictable events with a high economic and social impact [1]. Some researchers have defined the current health crisis as a black swan [2,3]. However, others, Taleb included, consider that this pandemic situation could have been predicted. In fact, experts in infectious disease/public health protection have been warning us for decades that a global pandemic involving a highly infectious respiratory disease virus was a plausible scenario [4,5,6].

There have been noteworthy precedents for other pandemics triggered by viruses or bacteria with a disastrous effect on human history [7,8,9,10,11]. Despite that, humanity has once again shown that it was not prepared to tackle these situations, even though being prepared for disasters can minimize damage to our health, lives, and property [12].

The current outbreak has been caused by severe acute respiratory syndrome coronavirus 2 (SARSCOV-2). Apart from the severe health problems [13], this pandemic has had a ripple effect on every aspect of human life as we know it, shaking our current society and affecting social, political and economic issues in a terrible way [3].

In this context, governments around the world have issued unprecedented policies and guidelines to save lives by reducing the pace and extent of COVID-19 infections (“flatten the curve”) [14,15]. Lockdown has been one of the most common measures in the beginning of the pandemic in a large number of countries, and it is considered the best public health containment strategy available [16]. Without exception, national lockdowns throughout the world have caused considerable disruption to individuals, families, households, communities, national economies, and societies as a whole [17,18] and in most countries, governments announcing lockdowns has led to the panic buying of food as well as premature shortages of goods and services [3,19,20,21].

Food is a basic need, and preserving the supply is essential during crises. The identification of the most influential variables on consumer behaviour is essential for companies in order to satisfy the demands of increasingly sophisticated and demanding food consumers [22]. Until now, different researchers developed models to explain consumer behaviour in the food selection process [23,24,25], which have been widely implemented.

Nevertheless, despite the common patterns of consumption being well known, the extraordinary situation that mankind has found itself immersed in is not conventional at all. Classic behavioural theories explaining consumer behaviour may not apply in this context [26].

The goal of this study was to analyse the food buying changes that have taken place in Spain in two time periods, with the reference point separating them being the declaration of state of alarm on 14 March 2020 due to the health crisis caused by COVID-19. The first time period is the week just before the state of alarm was declared, and the second is the first week after said declaration (lockdown), in order to compare them to regular buying behaviours.

In addition, the study also sought to learn the profile of food consumers, identifying those who are more sensitive to stressful situations and will change their regular buying behaviour the most, therefore being more susceptible to go panic buying.

With knowledge of the buying behaviour and profile of consumers in black swan situations (such as the COVID-19 pandemic, for example), agri-food companies will be able to plan ahead with business innovation strategies that allow them to, on one hand, decrease the troublesome behaviours of consumers, and on the other, guarantee the market supply in foreseeable economic, social, and health crises.

### 1.1. Panic Buying

Faced with the “fear of the unknown”, consumers took precautionary actions, stockpiling essential products to mitigate the risk of a possible stock shortage, which is often called “panic buying” [27]. The term is defined as the phenomenon that occurs when consumers buy unusually large amounts of products in anticipation of, during or after a disaster or perceived disaster, or in anticipation of a large price increase or shortage. It is a specific herd behaviour that is mainly triggered by a disaster or health crisis [26].

It has the potential to disrupt the supply chain with increased demand [27]. This disruption leads to more panic buying, thus creating a vicious circle. Moreover, panic buying reduces supply and creates higher demand, leading to higher price inflation. This increase in demand leads to a shortage of the product.

There are three mechanisms that can cause panic buying [28]. Firstly, it could be a manifestation of conflict between the desire to maintain routines versus uncertainty regarding the duration of the pandemic limiting access to daily necessities. Secondly, when a high risk is perceived, it is more likely for consumers to carry out panic buying in order to minimize their stress. In this regard, panic buying can be viewed as a self-protection mechanism to satisfy their safety needs [26]. Lastly, it could be a reaction in response to one’s loss of control of the future, causing people to react by conducting social behaviours that are similar to that of other consumers.

The recent cases of panic buying were not a novelty. There are previous pandemics or natural disasters such as earthquakes or hurricanes, that have prompted waves of panic buying [29,30,31,32].

### 1.2. The Food Attributes and Situational Factors That Influence Panic Buying

Several approaches have been adopted to model the buying behaviour of food consumers. Of these, the multi-attribute approaches are based on the assumption that quality is a multidimensional phenomenon [33]. Intrinsic cues are those that are associated with the physical properties of the product such as taste and flavour, whereas extrinsic attributes are all others, such as brand name and the reputation of the seller [34].

When panic buying, the importance that consumers assign the different product attributes is modified [35]. Thus, quality properties are usually less important than the amount [2], and consumers tend to be more accepting of high prices for the products [36], as long as they can secure the supply.

The situational factors that often affect shopping are altered [2]. Thus, the information transmitted by “reliable” sources is considered “cheap talk” and sometimes contributes to increase panic, meaning that the most “credible” information can be people’s experience when seeing empty shelves. Authors suggest that there can be two types of problems with this type of buying: coordination failure (all consumers buy at the same time) and information failure (consumers do not know the supply chain and they believe that the shortage can last a long time). Furthermore, in this digital era, information is readily available and can be quickly disseminated to masses over multiple channels that are also susceptible to abuse [26].

On the other hand, consumers’ buying decisions are often influenced by the choices of their peers, and this is more obvious in panic buying [37] where substitute products can be more readily accepted [38].

Panic buying is also affected by the measures that, both to mitigate stockpiling as well as to decrease the risk of contagion, may have been implemented at the places of purchase [39]. Among the measures established to prevent stockpiling are limiting the items that a single person can buy, shorter opening hours and information campaigns to deter stockpiling. Furthermore, to prevent possible contagion in the establishment, the most noteworthy measures are the distance between people, separation with screens, the recommendation to go shopping alone, following signs on the ground that keep the person shopping from going backwards, paying with credit cards, or distributing cleaning and protection material among the customers [29].

One of the situational factors that most affects buying and which has been modified by the implemented measures, is the time that can be allocated to it. Thus, it has been verified that time pressure changes the factors that affect buying food [39]. Shoppers under time pressure are less likely to make unplanned purchases compared to those who are not under time pressure [40], and this factor is an important determinant of aspects such as reading labels [41]. Under time restrictions, consumers have more trust for high-priced products and high-quality brands [42].

Furthermore, new technologies have really boomed during this crisis, and as well as enabling access to different sources of information [43], they have also modified distribution channels. The online channel, which had hardly been used for food until now, has become popular [20]. Moreover, it has been a very useful tool that limits the physical contact which is common when shopping [44].

### 1.3. The Consumer and Panic Buying

The panic buying behaviour does not affect all consumers equally. In general, it increases if consumers have previously experienced similar issues, and it decreases as the stock builds up [45]. Having experienced these issues previously is not essential however, as the effect of observing what happens to others is decisive [46].

It has also been observed that people with high levels of anxiety can go panic buying more often and stockpile more products. However, people with low levels of anxiety can also be dangerous because they are less likely to conduct the necessary actions to contain the pandemic [47]. Fear motivates people to go shopping because it gives them a sense of security and it alleviates their stress. It is a way of keeping their negative emotions under control [48,49].

Panic buying happens after numerous personal decisions in a short amount of time, which makes them especially difficult to research. However, it is important to do so because they can have a very negative effect. Panic buying is troublesome, and its consequences mainly affect vulnerable groups of people who cannot access essential goods such as food or water [3,26,27].

Furthermore, they also affect companies in the sector and supply chains. The food sector faced an increased demand due to the panic-buying and stockpiling of food. Disruptions have been minimal thus far, as food supply has been adequate, and markets have been stable. However, we have already seen challenges in terms of logistic bottlenecks, and there is likely less food of high-value commodities (i.e., fruits and vegetables) being brought to the market [50]. The supply of some products has been affected, forcing food companies and distribution chains to make significant efforts.

## 2. Materials and Methods

### 2.1. Database

For this study, Spanish buyers were surveyed using social networks Facebook and WhatsApp between 2 and 14 April 2020. It is a non-discriminatory exponential snowball sampling. The characteristics of the study and the state of alarm in Spain made it difficult to apply other sampling methods. A single questionnaire was submitted to each consumer during the lockdown, posing questions linked to the pre-lockdown week as well as the first week of the lockdown. The different sections that were in the questionnaire are listed in the methodology section.

We collected 528 valid responses. A confidence interval of 95.5% (*k* = 2) was selected, as this value is the most common one used in socioeconomic studies. The sample error was calculated within an infinite population (total food consumers in Spain) and assuming maximum indetermination (as it is usually done, with *p* = *q* = 0.5).

Before the fieldwork, a preliminary questionnaire was sent to 10 consumers to confirm that the questions of the survey were well designed and easily understandable.

A questionnaire with five parts was designed. The first included general buying habits before COVID-19. The second included questions on the level of concern and information on the health crisis. In the third, the questions addressed buying food the week prior to the state of alarm (7 to 13 March), and in the fourth, buying food during the first week of lockdown (14 to 20 March). Lastly, Section 5 includes the socio-economic data of the people polled, such as their gender, age, level of education, net monthly family income, and location of residence. The questionnaire used is included in the Supplementary File.

**Figure 1:**
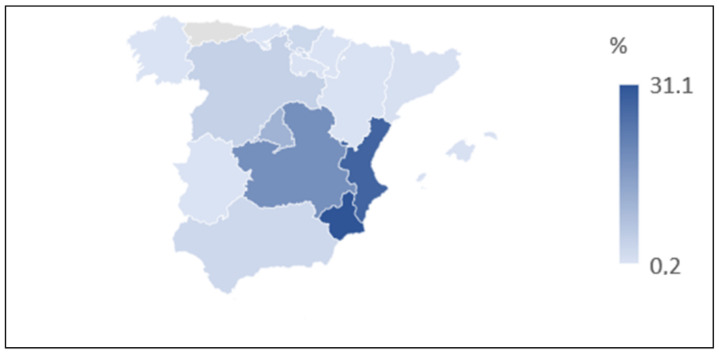
Sample distribution by autonomous community of origin.

Answers from almost all the autonomous communities of Spain were gathered, except for the Canary Islands and Asturias. However, the answers from the communities of Murcia, the Valencian Community, and Castile-La Mancha stand out because, by using snowball sampling, it has been affected by the location of origin of the researchers, who posted the questionnaire on their social networks. Figure 1 shows the distribution of the sample according to the community of origin.

In general, the people in charge of household shopping are mainly characterised by being women aged 35 to 49, with university studies, net monthly family incomes between €1000 and €1999 and who live in areas of high urban population concentration (Table 1).

### 2.2. Statistical Analysis

A segmentation of the sample population was conducted to analyse the information. The variable selected for segmentation was the variation of the importance of purchase attributes in this pandemic situation. To create this new variable, the importance that every individual consumer gave to the perceived importance of different food purchasing attributes in the pre-lockdown week and during the lockdown were compared. The attributes included were price, origin, place of purchase, type and size of packaging, commercial brand, organic certification, and designation of origin. The people polled were required to rate the importance attributed to the reported purchase attributes on a 5-point Likert scale, with 1 being the least important and 5 the most important in their day-to-day shopping. With that information, a new variable was established for each attribute, with three possible scores depending on the amount it changed: 1, if it did not change (a 3 rating on the Likert scale); 2, if it changed slightly (rating of 2 and 4 on the Likert scale, slightly less and slightly more, respectively); and 3, if it changed significantly (rating of 1 and 5 on the Liker scale, a lot less and a lot more, respectively). In this regard, three segments were identified in accordance with the variation in the assessment of purchase attributes of consumers due to the pandemic. Segment 1: No change in purchase attributes; Segment 2: Slight change in purchase attributes; and Segment 3: Major change in purchase attributes.

Previously, other studies have used the purchase attribute assessment to determine consumer segments [51,52,53]. Only extrinsic attributes have been considered in this case, as the study asks about food in general, and not a single product. Extrinsic attributes are product-related attributes which are not a part of the physical product, meaning they can be changed without altering the properties of the physical product [34]. Selecting these attributes was done by taking into account those which generally have a greater influence when buying food and which are most susceptible to be modified by panic buying. The attached table shows a summary of the extrinsic attributes taken into account and their consideration in other research papers (Table 2).

In order to look for differences among segments, we created a cross-tabulation table with a column proportions test for nominal variables and an Anova test to analyse scale and ordinal variables. The statistical tool used to contrast the hypothesis of independence between categorical variables was Pearson’s chi-squared test. Specifically, the cross-tabulation tables and chi-squared test were used to analyse the existence of significant differences between segments regarding different variables.

Results were analysed using the Statistical Package for Social Sciences IBM SPSS version 25.

## 3. Results and Discussion

The pandemic changed the importance that some consumers gave to different food purchasing attributes. Our segmentation identifies three different groups of consumers attending to the importance of those changes. The first segment, with 38.6% of the sample, included consumers who had not modified the importance assigned to the food purchasing attributes before and during the lockdown. The second segment, with 47.5% of the sample was comprised by consumers who showed slightly modifications. Finally, the third segment of consumers, with 13.9% of the sample, included those who showed severe changes.

### 3.1. Amount Purchased of Different Food Items

The variation of the amounts of different food items purchased by the consumer segments the week prior and after the state of alarm are shown on Table 3. This table shows the average scores for the amounts purchased before the state of alarm compared to those purchased in a normal situation. Thus, a one means that they buy much less than usual and a five means that they buy a lot more than usual. Therefore, scores over three indicate that more has been bought, and lower scores indicate that less has been bought.

In general, the food items purchased the most in the week prior to the state of alarm were rice, pasta and legumes, dairy products, meat, fresh fruit and/or vegetables, baked goods, and frozen foods. These are basic necessity foods and many of them are easily storable. Of these, in week 1 of the lockdown, the increase in dairy products, meat, fruit and vegetable and baked goods remained the same and the purchase of rice, pasta and legumes, and frozen products fell. The least purchased food items before the lockdown were soft drinks and juices, spices, condiments and sauces, snacks, bottled water, and beer, wine, and spirits. Of these, the purchase of beer and snacks increased in week 1. The purchase of bottled water and soft drinks remains the same. Lastly, the foods that remained the same before the lockdown are coffee and infusions, olive oil, fish, and canned products. All of the latter fell in week 1 of the lockdown. Regarding olive oil, an essential part of the Mediterranean diet, it was not added in a significant way when panic buying before the state of alarm, as all segments have ratings of around 3.

In segment 1, which comprised 38.6% of consumers, the most purchased foods in the pre-lockdown week were the same as for the total population, although the amount purchased was slightly lower in all cases: dairy products, baked goods, meat, fruit, rice, pasta, and legumes. Of these, in week 1 of the lockdown, only the purchase of meat increased, whereas rice, pasta and legumes decreased, with this fall being more significant than for other segments. Of the least bought products, snacks and beer, their purchase increased the following week, and it did so in greater proportion than for the total population. For the foods that remained the same, the amount purchased decreased in week 1 of the lockdown for oil, fish, and canned food, whereas for coffee it remained similar.

In segment 2, which comprised 47.5% of consumers, the food items that were purchased the most were similar to the total population, and the amounts purchased were slightly higher than the total population. The variation in week 1 of the lockdown was also similar. Regarding the products whose amount purchased dropped, they were also similar, and in week 1 the only group that increased were snacks, with the amount purchased for all other products remaining the same. For the food items that remain more stable, the only group that was purchased less was canned food, with all other categories remaining the same. This is the intermediate segment, which reacted by modifying the amounts purchased, but in a more moderate way than segments 1 and 3.

Lastly, segment 3, which comprised 13.9% of consumers, is the most sensitive to change. The increase in the amount purchased the week prior to the lockdown took place for the same groups of products as for the total population, but in this case, the increase was more pronounced. In week 1 of the lockdown, the amounts purchased were similar for all groups of products, except for rice, pasta, and legumes. The products purchased at a lesser rate were similar to the rest of the population, but it is worth noting that the purchase of beer, wine, and snacks was much lower during the week prior, and these levels were maintained in week 1 of the lockdown. This segment also purchased less spices, condiments, and sauces, and during week 1 they purchased even less of these. In the categories of more stable products, there was a decrease in the amounts of coffee, oil, and canned food purchased. The amount of fish purchased remained lower than normal but stable during that period of time.

### 3.2. Food Buying and Places of Purchase

As regards shopping, a majority of consumers leave the home to do it, but this proportion was noticeably lower in segment 3, where the online or telephone option was much more prominent than for the other segments. When asked if they conducted these ways of shopping before COVID-19, the answers were very similar in all segments, and the percentage of those who did it often or on a regular basis was very low. However, this crisis boosted these ways of shopping, especially among consumers in segment 3. However, when asked whether they will continue using online shopping, in this segment there was a smaller percentage of consumers that will do so compared to others, where almost 30% of consumers said they will use the online channel to buy food. These results can be found in Table 4.

Regarding the places of purchase (Table 5), the scale used was an ordinal scale of five items, where 1 means that the person never purchased at that type of establishment, and a five means that they always did. The establishment of choice was the supermarket or hypermarket, followed by traditional shops, indoor markets and, lastly, buying straight from the producer, and there were hardly any differences among segments. Regarding the differences in behaviour between the week prior to the lockdown and during the lockdown, there were differences in almost all cases. The supermarket/hypermarket as the preferred choice increased mainly for segment 1, whereas it decreased for segment 3, although not significantly. All other places of purchase suffered a drop in importance.

### 3.3. Purchase Attributes

**Figure 2:**
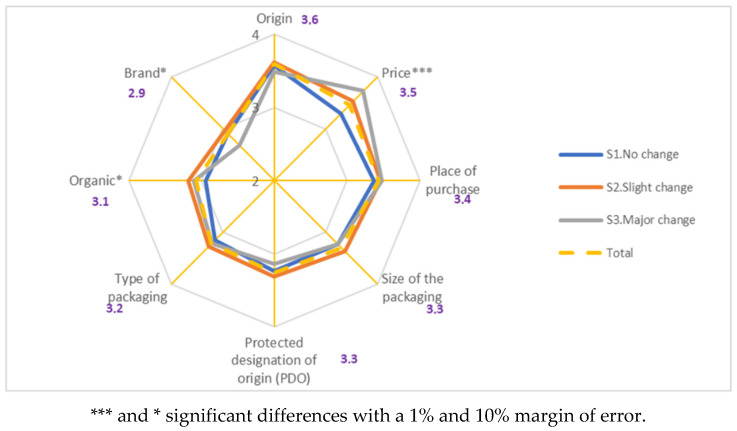
Attribute value by consumer segments.

The most highly valued attribute was the origin, followed by the price, place of purchase, size of the packaging, the protected designation of origin (PDO) label, the type of packaging, and the organic certification of the food. The brand proved to be less important. The attributes that show significant differences between the consumer segments were the price, brand, and organic nature of the product. Consumers in segment 1 valued the price less and the organic nature slightly less, whereas, regarding the brand, they were in line with the rest of the population. Segment 2 consumers valued the organic nature and brand more, whereas those in segment 3 stood out for assigning greater value to the price, and less to the brand (Figure 2).

**Figure 3:**
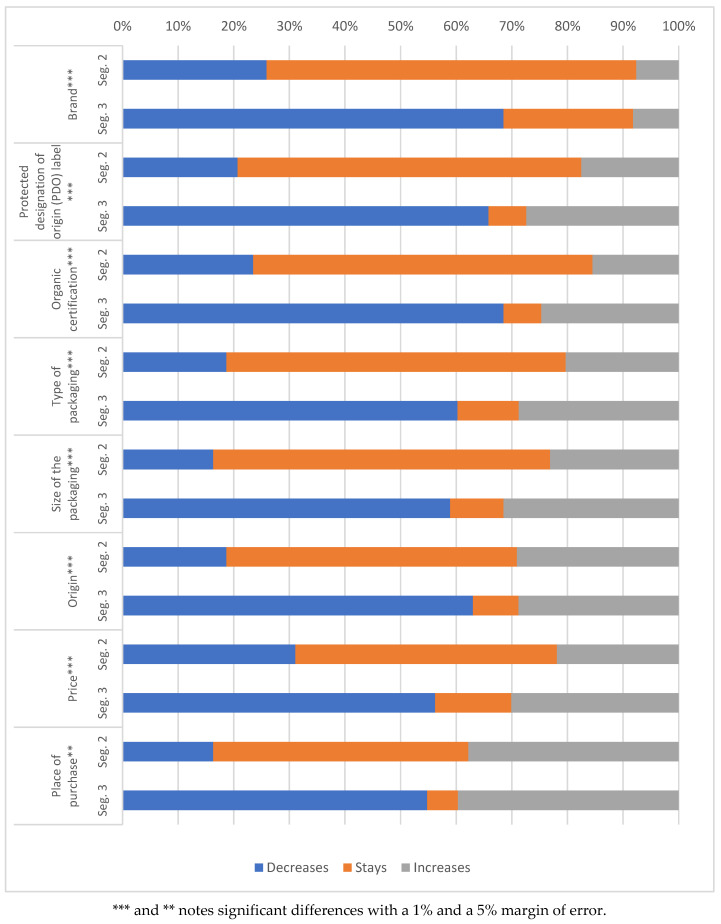
Change in the value assigned to the attributes.

Regarding the direction of the change in importance assigned to the attributes, segment 1 has not been considered, as it did not modify the value it assigns to the attributes (Figure 3). In general, in segment 3 there was a greater decrease in the importance assigned to purchase attributes.

If we analyze them individually, the brand is the attribute that most decreased in importance, especially for segment 3. However, regarding the designation of origin, although its decrease was also majoritarian, there was a 24.7% of consumers in segment 3 who assigned it greater importance after the lockdown. This also happened with organic certification.

The place of purchase was the attribute with the most significant increase. In segment 2, there was a high percentage of people who maintained its importance, and in segment 3, compared to the other attributes, its importance decreased for a smaller percentage of consumers.

The size of packaging is the second attribute for which a greater proportion of consumers increase its importance. Regarding the origin, there was a large percentage of people in segment 3 who said its importance decreases. However, in segment 2, the percentage of people who assigned it the same level of importance was very high.

The price is the attribute that most decreased in importance for segment 2, whereas for segment 3, there was a greater percentage of people who maintained its importance compared to the other attributes.

### 3.4. Level of Concern for COVID, Impact, and the Search for Information

The level of concern was very high and there were no significant differences in this variable (Table 6). Regarding the impact, it was fairly negative for all segments, especially for segment 3. Consumers in segment 1 were more optimistic.

The results reveal a high concern for the pandemic, and only 1.5% of consumers were not interested in the information. Consumers in segment 3 were more concerned and also spend more time seeking information, especially through the news programs on TV and official sources. The opinion of friends and family and social networks are less important, although they are taken into account by segment 2 (Table 7),

### 3.5. Socio-Economic Characteristics of People Polled by Segments

Only the socio-economic characteristics with significant differences among segments are shown (Table 8). Only differences in age and family size emerged. Regarding age, in segment 3 there was a larger of percentage of younger consumers (aged 18 to 34). The segment of people whose behaviour did not change has a smaller family size. Regarding gender and work activity there were no significant differences.

## 4. Discussion

The results of the survey have made it possible to learn how food consumers behaved right before the lockdown and in the first week of the lockdown in Spain, and which consumer profiles changed their behaviour the most. This section analyses the results taking other research into account.

In our study, 61.4% of consumers modified their buying behaviour. Other studies, such as one conducted in China after the COVID-19 outbreak, showed that the pandemic had a psychological impact on 54% of consumers [108]. People with higher levels of anxiety can conduct panic buying more often and stockpile more products. This can have a harmful effect on the community, which may need these resources for other purposes. On the other hand, people who modify their behaviour less can also be dangerous because they are less likely to conduct the necessary actions to contain the pandemic [47].

During the period of time analysed, the amounts purchased have changed. This can be due to the fact that consumers react when they believe that products will be scarce in order not to have their ability to choose limited [26]. Furthermore, other authors say that an increase in shopping can decrease stress before an unknown situation [48,49].

Stockpiling products the week before the lockdown also occurred in other countries, but it was slightly different. In Italy, the storable products that were in plastic packaging, which are perceived as being safer, were also bought more often [19]. However, the purchase of fresh products decreased, which did not happen in Spain. Concern for a healthy diet may be behind this behaviour [43]. However, the purchase of rice, pasta, and legumes and frozen products fell, possibly due to the fact that they were already stored in the homes. On the other hand, there were products that were purchased less in the week before the lockdown, but which were purchased more during the lockdown, such as beer or snacks. These are products that were usually consumed in bars or restaurants and are now consumed at home, and it may be because their consumption is linked to their symbolic value and the tendency to continue some external socialisation habits at home [19]. In our segments, segment 3 (panic buyers) is the one that buys the least snacks, beer, and wine, meaning it has not transferred social activities to the household or has not made prize product purchases. On the contrary, segment 1 modified its behaviour the least, except for beer and snacks, meaning it could be the segment that, while experiencing the least amount of alarm, needed to take its socialisation habits home the most. On the other hand, beer and snacks are products that can be considered “prize products”, whose purchase increases as a result of a disaster [32]. Further noteworthy, is the drop in the purchase of olive oil in the first week of the lockdown, which could be due to it being a product with a high caloric value and consumers trying to decrease its intake [43].

Regarding the places of purchase, online purchasing, which until now had been a seldom used channel by food buyers, shows a significant increase at the expense of the conventional channels. This result is in line with those obtained in studies conducted on previous crises [109,110,111] as well as more recent ones focused on the COVID crisis [19,20]. The loss of importance of all other purchasing establishments has also been documented in other countries such as Italy [19].

In general, food attributes have fallen in importance, especially for panic buyers, which can be in line with the studies that say that, when panic buying, substitute products are more acceptable, in other words, consumers are more content with the products they find and do not carry out “specific searches” [38]. When analysing the evolution of these attributes individually, it is observed that the place of purchase of the attribute that has increased its importance for a greater proportion of consumers. This can be linked to other attributes derived from shopping. In general, consumers seek less contact, which is why they try to go shopping less often and seek safe places to do so [111]. During the two periods of time analysed, there is a loss of importance of the brand as a purchase attribute. Even though this contradicts one study [42], can be in line with another [38], which reveals the better acceptance of substitutes in panic situations. On the other hand, both the designation of origin and the organic label increased in importance during the lockdown for a significant percentage of consumers. It could be that established brands, which are well positioned regarding the perception of quality of their products, work better than private brands, and that the result is in line with some authors [29], which says that well-positioned brands will have an advantage during pandemics. Furthermore, authors [112] says that in situations of risk, consumers prefer to buy organic products. The price, which decreased in importance for segment 2 and remained stable for segment 3, in other studies [42] was seen as an indicator of the quality of the product, meaning it is a significant attribute, while revealing that a high price increases consumer confidence. Furthermore, when a product shortage is predicted, the consumer tends to be more accepting of higher prices for products [36]. The changes in the importance assigned to the size of the packaging can also be linked to the lower frequency with which people go shopping [111]. On the other hand, if we take into account that people often go shopping in order to store the food, both the type of packaging and its size can be very important. Lastly, the decrease in importance of the origin attribute for panic buyers and its preservation among consumers in segment 2 are in line with the findings of other studies [20,111].

Regarding the level of concern for Covid, a study [112] believes that those consumers who perceive greater risks change their buying behaviours more, which has also been observed in our results (Table 7). However, the fact that consumers in segment 3, who perceived a greater risk, still consider the price important, can be in line with the consideration that price restrictions are a consequence of the foreseeable economic crisis [29].

Regarding the sources of information, media outlets play a very important role to provide credible information to consumers on how the supply chain works, and thus alleviate the issues derived from stockpiling [2] as well as favouring an increase in confidence among consumers in the public authorities, while possibly encouraging a return to the normal buying behaviour [46]. On the other hand, social networks and the opinion of friends and family, despite being less important in this study, is direct information that can have a great influence on behaviour [46]. How prepared people are for disasters should take into account the disastrous events that are likely to happen and what/who is likely to be affected in different parts of countries and cities [12]. This can be done with suitable information from official channels, but also using social networks, which are gaining traction as media outlets among consumers.

The results obtained regarding socioeconomical variables show that in the segment of panic buyers there is a greater proportion of young people, which is consistent with the fact that the COVID crisis has an effect regarding changing behaviour that is less significant among older people [112]. In segment 1, which includes those who experience less changes in their behaviour, the size of the family is smaller, which is in line with the concern of the consumers with their family becoming infected [108]. Lastly, in our study there were no differences regarding gender and work activity, whereas in another study there were differences in these variables, having a greater impact on women and students [108].

## 5. Conclusions

The work conducted has made it possible to detect three significant segments of consumers based on their food buying behaviour, which also made it possible to establish the variations experienced both regarding the place of purchase as well as the extrinsic attributes of the food items, as well as the profile of the consumers in both analysed periods.

Up to 61.4% of consumers modified their buying behaviour, with the consumers that most modified the value they assign to the attributes being those who stockpiled the most (panic buying), whereas those who changed it the least, shopped as usual (38.6%).

The most valued attributes were the origin, followed by price, place of purchase, size of packaging, protected designation of origin label, type of packaging, and organic certification of the food, in this order. It is worth noting the low importance that consumers assigned to the brand, maybe due to the fact that what was important was having supplies.

Regarding the attributes with significant differences among consumer segments, we see that whereas consumers in segment 1 (38.6% of consumers) assigned less value to the price and organic certification, and more to the brand, consumers in segment 2 (47.5% of consumers) assigned greater value to the organic certification and the brand. Lastly, consumers in segment 3 (13.9% of consumers) assigned greater value to the price and less to the brand.

In this sense, the study has verified that the importance assigned to purchase attributes has been modified to a greater or lesser extent, especially for the brand, which can indicate that the consumer has more readily accepted substitute products. However, attributes such as the protected designation of origin or organic certification, which are linked to quality assurance and food safety increased their importance, especially among consumers who were more prone to change, but not the price, which was different for each segment, gaining importance among consumers who were more prone to panic buying, possibly because they also perceived greater risk from the health, economic and social crisis.

Further noteworthy, is the change in place of purchase of the food, as it is observed that supermarkets and hypermarkets, where it is more feasible to find a broader range and in greater amounts for storing, benefited. In this sense, online food buying, which until before the pandemic had very limited importance, has now gained traction and many consumers expressed their willingness to continue using this way of shopping.

Lastly, it has been verified that the official channels of information and written press (in paper and online) are the most reliable sources of information that reach consumers and also modify their buying behaviour the most, whereas over social networks it is possible to reach consumers who have maintained a more stable buying behaviour.

The characterization of the profile of the different consumer segments can allow food production and/or distribution companies to implement different innovative, customized, and more effective strategies, aimed at decreasing the impact of stockpiling, and therefore of food shortage. To do so, the alternative strategies they can implement include a stock of non-perishable foods as a strategic reservoir in their warehouses in preparation for eventual increases in demand, to increase production capabilities in a sustainable way, and especially, to promote the online sale and distribution of food, with the objectives of lowering the amount of people in shops (which decreases the spreading of the pandemic and favours health) and preventing consumers from encountering possible circumstantial shortages that would only encourage stockpiling and panic buying, even among consumers who do not change their buying behaviour.

The goal was for the results of the research to discover the changes in buying behaviour of food consumers in situations of severe stress, due to the COVID-19 pandemic, compared to their regular behaviour. In turn, this situation has led to an economic and social crisis that only the income policy of the government has attempted to alleviate, in order to prevent it from becoming a financial and credit crisis.

Unfortunately, this crisis has shown countries, such as Spain and others in Europe that do not suffer major natural disasters like other countries in the world that routinely witness hurricanes, earthquakes, major fires, floods, droughts, epidemics, etc., that they must be prepared, as they are increasingly frequent in other parts of the world where they did not use to happen, having a significant impact on agricultural production, which is the basis for the production of foods.

Without a doubt, the increased frequency of natural disasters is caused by climate change, as has been revealed by the Intergovernmental Panel on Climate Change (IPCC). Furthermore, the transportation of people around different parts of the world has contributed to disseminate what were once local epidemics, making them global pandemics, as has happened this year with COVID-19, and the possibility that other pandemics may occur in the future cannot be ruled out..

Having said this, the goal of this study is not just to be prepared for future pandemics, but also to be vigilant for future situations that may occur and cause stress among consumers, panic buying, food stockpiling and, potentially, shortages. Being prepared is the responsibility of the private sector (from the standpoint of logistics), as well as the public authorities (from the standpoint of truthful information for the population). The ultimate goal is to care for the basic needs of the population, one of which is eating.

Only when ready is it possible to respond appropriately. What is very probable is that many of these changes in the buying behaviour of consumers will happen again in situations of health, economic, financial and/or social crises. Knowing and identifying when they take place will make it possible to plan for them in advance, making quick and useful decisions from the field of the agri-food sector.

## Figures and Tables

**Table 1:** Socio-economic characteristics of the sample of people who are in charge of shopping.

Variables	%
Gender	
Male	32.0
Female	68.0
Age (in Years)	
18–24	5.1
25–34	20.6
35–49	44.5
50–64	26.5
>65	3.3
Education Level	
Elementary	2.1
Secondary	13.6
University	84.3
Income	
<€1000	6.4
€1000–€1999	30.9
€2000–€2999	28.0
€3000–€3999	20.3
>€4000	14.4
Area of Residence	
Rural (<30,000 inhab.)	19.8
Urban (30,000–100,000 inhab.)	16.2
High urban population concentration (100,001–500,000 inhab.)	53.4
Metropolitan area (>500,000 inhab.)	10.6

**Table 2:** Extrinsic attributes considered in some food research studies.

Attribute	References
Price	[54,55,56,57,58,59,60,61]
Origin	[57,58,62,63,64,65,66,67,68]
Place of purchase	[69,70,71,72,73,74,75,76]
Type and size of packaging	[77,78,79,80,81,82,83,84,85]
Commercial brand	[55,59,86,87,88,89,90,91,92,93]
Organic certification	[54,55,64,86,92,94,95,96,97,98]
Designation of origin (guarantee label)	[63,76,87,99,100,101,102,103,104,105,106,107]

**Table 3:** Amount of different food groups purchased in the pre-state of alarm week and in which direction the change went in the first week post-state of alarm for each of the consumer segments.

Food	Seg. 1 No Change (38.6%) ^1^			Seg. 2 Slight Change (47.5%) ^1^			Seg. 3 Major Change (13.9%) ^1^			Total		
Rice, pasta, legumes ***	3.30	***	↓	3.52	**	↓	3.56	***	↓	3.44	***	↓
Dairy products **	3.18			3.36	**	↑	3.37			3.29	**	↑
Meat **	3.14	*	↑	3.28			3.38			3.24	*	↑
Fresh fruit and vegetables ***	3.10			3.27	***	↑	3.36			3.22	***	↑
Baked goods *	3.12			3.21	***	↑	3.29			3.18	***	↑
Frozen foods	3.08			3.11	*	↓	3.04			3.09	***	↓
Coffee and infusions	2.98			3.06			3.04	**	↓	3.03	***	↓
Olive oil	3.00	*	↓	3.02			2.96	*	↓	3.00	***	↓
Fish	2.99	**	↓	2.94			2.90			2.95	**	↓
Canned food	2.93	**	↓	2.98	***	↓	2.93	**	↓	2.95	***	↓
Beer, wine, and spirits ***	2.94	*	↑	2.93			2.59			2.89	**	↑
Bottled water	2.92			2.86			2.84			2.88		
Snacks *	2.90	*	↑	2.92	*	↑	2.64			2.88	**	↑
Spices, condiments, and sauces *	2.77			2.76			2.55	**	↓	2.74	**	↓
Soft drinks and juices ***	2.71			2.76			2.33			2.68		

^1^ Size of the segment; ***, ** and * indicate significant differences with a 1%, 5%, and 10% margin of error; the asterisks shown on the food column reflect differences among segments. The asterisks shown in the segment column refer to the differences between the week prior and the first week after the state of alarm for each segment; the arrows mark the direction of the variation ↓ the amounts purchased decreased in week 2 compared to week 1. ↑ The amounts purchased increased in week 2 compared to week 1.

**Table 4:** Traditional shopping versus online food shopping for home.

Food Shopping for Home	Seg. 1 (38.6%) ^1^	Seg. 2 (47.5%) ^1^	Seg. 3 (13.9%) ^1^	Total
Buying Food ***				
Yes, I leave home to shop	89.2% ^a^	87.3% ^a^	72.6% ^b^	86.0%
No, I make a list and a friend/relative brings it to me	7.8% ^a^	7.6% ^a^	9.6% ^a^	8.0%
No, I do it over the Internet	2.5% ^a^	3.2% ^a^	12.3% ^b^	4.2%
No, I do it over the telephone	0.5% ^a^	2.0% ^a,b^	5.5% ^b^	1.9%
Online Food Purchasing (Before the State of Alarm)				
Never	63.7% ^a^	61.0% ^a^	72.6% ^a^	63.6%
Rarely	18.6% ^a^	19.1% ^a^	8.2% ^b^	17.4%
Sometimes	15.2% ^a^	17.5% ^a^	17.8% ^a^	16.7%
Most of the time	2.0% ^a^	1.6% ^a^	0.0%	1.5%
Always	0.5% ^a^	0.8% ^a^	1.4% ^a^	0.8%
Online Food Purchasing (After the State of Alarm)	30.40% ^a^	35.10% ^a^	47.90% ^b^	35.00%
Consumers that Will Continue Buying Online				
Definitely not	33.9%	27.3%	45.7%	33.0%
I don’t know	37.1%	45.5%	40.0%	41.6%
Definitely will	29.0%	27.3%	14.3%	25.4%

^1^ Size of the segment; *** note significant differences with a 1% margin of error, respectively; Different letters in the same row mean significant differences for the segments (*p* < 0.05).

**Table 5:** Place of purchase of food for home.

Place of Purchase	Seg. 1 (38.6%) ^1^	Seg. 2 (47.5%) ^1^	Seg. 3 (13.9%) ^1^	Total
Supermarket/Hypermarket				
Pre-state of alarm	4.06 *	4.05	4.21	4.08
Post-state of alarm	4.24 *	4.06	4.03	4.12
*In Traditional Shops*				
Pre-state of alarm	2.94 *	3.13 *	3.14 *	3.06
Post-state of alarm	2.61 *	2.79 *	2.71 *	2.71
*In Indoor Markets/Markets*				
Pre-state of alarm ***	2.15 *	2.36 *	2.10 *	2.24
Post-state of alarm	1.50 *	1.73 *	1.62 *	1.63
*Directly from the Producer*				
Pre-state of alarm	1.39 *	1.38 *	1.34	1.38
Post-state of alarm	1.16 *	1.21 *	1.27	1.20

^1^ Size of the segment; *** and * note significant differences with a 1% and 10% margin of error, respectively; the asterisks in the segment boxes refer to the change between considered periods (*t*-test of related samples). The asterisks on the variables column refer to the differences between segments (ANOVA).

**Table 6:** Level of concern and impact of the COVID-19 crisis in Spain.

Level of Concern and Impact of the COVID-19 Pandemic	Seg. 1 (38.6%) ^1^	Seg. 2 (47.5%) ^1^	Seg. 3 (13.9%) ^1^	Total
Level of Concern				
Not at all concerned	1.0%	0.0%	1.4%	0.6%
Slightly concerned	2.5%	0.8%	1.4%	1.5%
Somewhat concerned	14.2%	9.6%	8.2%	11.2%
Moderately concerned	32.4%	36.7%	28.8%	33.9%
Extremely concerned	50.0%	53.0%	60.3%	52.8%
Impact on the Family Economy ***				
Very negative	13.7% ^a^	24.3% ^b^	35.6% ^b^	21.8%
Somewhat negative	55.4% ^a^	47.0% ^b^	37.0% ^b^	48.9%
Neutral	27.9% ^a^	23.1% ^a^	20.5% ^a^	24.6%
Somewhat positive	2.5% ^a^	4.8% ^a^	4.1% ^a^	3.8%
Very positive	0.5% ^a^	0.8% ^a^	2.7% ^a^	0.9%

^1^ Size of the segment; *** notes significant differences with a 1% margin of error; Different letters in the same row mean significant differences for the segments (*p* < 0.05).

**Table 7:** Time spent seeking information on COVID-19 (h/day) and preferred sources of information.

Time and Sources of Information	Seg. 1 (38.6%) ^1^	Seg. 2 (47.5%) ^1^	Seg. 3 (13.9%) ^1^	Total
Time Spent Seeking Information (hrs/day)				
I’m not interested in being informed	2.5% ^a^	1.2% ^a^	0.0%	1.5%
Less than 1 h	37.3% ^a^	35.9% ^a^	35.6%^a^	36.4%
Between 1 and 2 h	44.6% ^a^	40.6% ^a^	34.2% ^a^	41.3%
Between 2 and 4 h	9.8% ^a^	15.5% ^a,b^	21.9% ^b^	14.2%
More than 4 h	5.9% ^a^	6.8% ^a^	8.2% ^a^	6.6%
*Sources of Information*				
TV news ***	3.49	3.70	3.36	3.57
Newspapers (in paper or online)	3.29	3.49	3.45	3.41
Relatives and/or friends	3.11	3.22	2.99	3.15
Official sources ***	2.95	3.18	3.29	3.10
Social networks	2.97	3.14	2.93	3.05

^1^ Size of the segment; *** notes significant differences with a 1% margin of error; Different letters in the same row mean significant differences for the segments (*p* < 0.05).

**Table 8:** Age and family size of the consumer segments by their assessment of the purchase attributes.

Age and Family Size	Seg. 1 (38.6%) ^1^	Seg. 2 (47.5%) ^1^	Seg. 3 (13.9%) ^1^	Total
Age (in Years) **				
18–24	5.4% ^a,b^	3.6% ^b^	9.6% ^a^	5.1%
25–34	19.6% ^a^	17.9% ^a^	32.9% ^b^	20.6%
35–49	44.6% ^a^	48.6% ^a^	30.1% ^b^	44.5%
50–64	25.5% ^a^	27.9% ^a^	24.7% ^a^	26.5%
>65	4.9% ^a^	2.0% ^a^	2.7% ^a^	3.2%
Family Size (members) *	2.84	3.12	3.01	2.99

^1^ Size of the segment; ** and * note significant differences with a 5% and 10% margin of error, respectively; Different letters in the same row mean significant differences for the segments (*p* < 0.05).

## Supplementary Materials

The following are available online at https://www.mdpi.com/2304-8158/9/12/1821/s1, Supplementary File: Food buying habits during a crisis: Covid-19.
