# Supplementary material for: Innovation Strategies of the Spanish Agri-Food Sector in Response to the Black Swan COVID-19 Pandemic
Source: Foods. 2020 Dec 8;9(12):1821. doi: 10.3390/foods9121821 (PMC7762556; doi:10.3390/foods9121821)
Supplement: Supplementary file 1 [file foods-09-01821-s001.pdf]

## Food buying habits during a crisis: Covid-19

A group of researchers from the University Miguel Hernández of Elche and the University of Castilla-La Mancha are carrying out a study on purchasing habits in times of crisis. If you are so kind, we would like you to take a few minutes of your time to complete this questionnaire. The information obtained will be treated confidentially. Thank you very much for your collaboration.

1. Are you the person responsible for shopping in your home?
☐ Yes ☐ No (end of the survey)

**ABOUT YOUR GENERAL PURCHASE HABITS**

*To answer the questions in this section, think about your shopping habits before the COVID-19 crisis*

2. How often do you usually buy food from the following places of purchase?

| | 1 Never | 2 | 3 | 4 | 5 Always |
|---|---|---|---|---|---|
| Supermarket/hypermarket | ☐ | ☐ | ☐ | ☐ | ☐ |
| In traditional shops | ☐ | ☐ | ☐ | ☐ | ☐ |
| In indoor markets/markets | ☐ | ☐ | ☐ | ☐ | ☐ |
| Directly from the producer | ☐ | ☐ | ☐ | ☐ | ☐ |

3. How important are the following attributes to you when you buy food?

| | 1 Not at all important | 2 | 3 | 4 | 5 Very important |
|---|---|---|---|---|---|
| Price | ☐ | ☐ | ☐ | ☐ | ☐ |
| Brand | ☐ | ☐ | ☐ | ☐ | ☐ |
| Origin | ☐ | ☐ | ☐ | ☐ | ☐ |
| Place of purchase | ☐ | ☐ | ☐ | ☐ | ☐ |
| Type of packaging | ☐ | ☐ | ☐ | ☐ | ☐ |
| Size of packaging | ☐ | ☐ | ☐ | ☐ | ☐ |
| Organic label | ☐ | ☐ | ☐ | ☐ | ☐ |
| Designation of origin | ☐ | ☐ | ☐ | ☐ | ☐ |

4. Prior to the COVID-19 crisis, had you ever purchased food over the Internet?

| 1 Never | 2 | 3 | 4 | 5 Always |
|---|---|---|---|---|
| ☐ | ☐ | ☐ | ☐ | ☐ |

**ABOUT YOUR LEVEL OF CONCERN AND INFORMATON ON THE HEALTH CRISIS**

5. Please indicate your level of concern with the coronavirus health crisis (COVID-19)

| 1 Not at all concerned | 2 | 3 | 4 | 5 Very concerned |
|---|---|---|---|---|
| ☐ | ☐ | ☐ | ☐ | ☐ |

6. Indicate the impact of the crisis in your family economy

| 1 Very negative | 2 | 3 | 4 | 5 Very positive |
|---|---|---|---|---|
| ☐ | ☐ | ☐ | ☐ | ☐ |

7. Please indicate how often you are using the following sources of information on the health crisis

| | 1 Never | 2 | 3 | 4 | 5 Always |
|---|---|---|---|---|---|
| Official sources | ☐ | ☐ | ☐ | ☐ | ☐ |
| TV news | ☐ | ☐ | ☐ | ☐ | ☐ |
| Newspapers (paper/online) | ☐ | ☐ | ☐ | ☐ | ☐ |
| Radio | ☐ | ☐ | ☐ | ☐ | ☐ |
| Friends and relatives | ☐ | ☐ | ☐ | ☐ | ☐ |
| Social networks | ☐ | ☐ | ☐ | ☐ | ☐ |

8. How much time did you spend yesterday seeking information about the health crisis?

| I'm not interested in being informed | Less than 1 hour | Between 1 and 2 hours | Between 2 and 4 hours | More than 4 hours |
|---|---|---|---|---|
| ☐ | ☐ | ☐ | ☐ | ☐ |

**ABOUT YOUR FOOD PURCHASE IN THE WEEK BEFORE THE STATE OF ALERT**

*We are now going to ask you about your purchasing behaviour in the week before the state of alert was declared (before 16 March)*

9. In the week before the lockdown, indicate how much you bought the following foods compared to your usual purchase.

| | 1 Less than usual | 2 | 3 | 4 | 5 More than usual |
|---|---|---|---|---|---|
| Dairy products | ☐ | ☐ | ☐ | ☐ | ☐ |
| Baked goods | ☐ | ☐ | ☐ | ☐ | ☐ |
| Meat | ☐ | ☐ | ☐ | ☐ | ☐ |
| Fish | ☐ | ☐ | ☐ | ☐ | ☐ |
| Rice, pasta, legumes | ☐ | ☐ | ☐ | ☐ | ☐ |
| Bottled water | ☐ | ☐ | ☐ | ☐ | ☐ |
| Soft drinks and juices | ☐ | ☐ | ☐ | ☐ | ☐ |
| Beer, wine and spirits | ☐ | ☐ | ☐ | ☐ | ☐ |
| Canned food | ☐ | ☐ | ☐ | ☐ | ☐ |
| Frozen foods | ☐ | ☐ | ☐ | ☐ | ☐ |
| Coffee and infusions | ☐ | ☐ | ☐ | ☐ | ☐ |
| Spices, condiments, sauces | ☐ | ☐ | ☐ | ☐ | ☐ |
| Olive oil | ☐ | ☐ | ☐ | ☐ | ☐ |
| Snacks | ☐ | ☐ | ☐ | ☐ | ☐ |

**ABOUT YOUR FOOD PURCHASE IN THE LOCKDOWN**

*In this section, we will ask you about your purchasing behaviour in the current confinement period*

10. Are you going out to buy food for your home?

| Yes, I leave the house to do the shopping | No, I do the shopping online | No, I make a shopping list and a family member or friend brings it home | No, I do the shopping by phone |
|---|---|---|---|
| ☐ | ☐ | ☐ | ☐ |

11. Once the period of confinement is over, do you think you will continue to buy food online?

| I have not done any purchase online | I'm sure I won't | I don't know | I'm sure I will |
|---|---|---|---|
| ☐ | ☐ | ☐ | ☐ |

12. During the period of confinement, indicate how often you are buying food from these places

| | 1 Never | 2 | 3 | 4 | 5 Always |
|---|---|---|---|---|---|
| Supermarket/hypermarket | ☐ | ☐ | ☐ | ☐ | ☐ |
| In traditional shops | ☐ | ☐ | ☐ | ☐ | ☐ |
| In indoor markets/markets | ☐ | ☐ | ☐ | ☐ | ☐ |
| Directly from the producer | ☐ | ☐ | ☐ | ☐ | ☐ |

13. How many times have you gone to buy food during the lockdown compared to your usual frequency?

| Much less than usual | Less than usual | The usual amount | More than usual | Much more than usual |
|---|---|---|---|---|
| ☐ | ☐ | ☐ | ☐ | ☐ |

14. During this last week, indicate how much you bought of the following foods compared to your usual purchase

| | 1 Less than usual | 2 | 3 | 4 | 5 More than usual |
|---|---|---|---|---|---|
| Dairy products | ☐ | ☐ | ☐ | ☐ | ☐ |
| Baked goods | ☐ | ☐ | ☐ | ☐ | ☐ |
| Meat | ☐ | ☐ | ☐ | ☐ | ☐ |
| Fish | ☐ | ☐ | ☐ | ☐ | ☐ |
| Rice, pasta, legumes | ☐ | ☐ | ☐ | ☐ | ☐ |
| Bottled water | ☐ | ☐ | ☐ | ☐ | ☐ |
| Soft drinks and juices | ☐ | ☐ | ☐ | ☐ | ☐ |
| Beer, wine and spirits | ☐ | ☐ | ☐ | ☐ | ☐ |
| Canned food | ☐ | ☐ | ☐ | ☐ | ☐ |
| Frozen foods | ☐ | ☐ | ☐ | ☐ | ☐ |
| Coffee and infusions | ☐ | ☐ | ☐ | ☐ | ☐ |
| Spices, condiments, sauces | ☐ | ☐ | ☐ | ☐ | ☐ |
| Olive oil | ☐ | ☐ | ☐ | ☐ | ☐ |
| Snacks | ☐ | ☐ | ☐ | ☐ | ☐ |

15. Next, we ask you about the aspects that are taken into account when buying food. Could you indicate if you value them more, the same or less than before the COVID-19 crisis?

| | 1<br>Not at all important | 2 | 3 | 4 | 5<br>Very important |
|---|---|---|---|---|---|
| Price | ☐ | ☐ | ☐ | ☐ | ☐ |
| Brand | ☐ | ☐ | ☐ | ☐ | ☐ |
| Origin | ☐ | ☐ | ☐ | ☐ | ☐ |
| Place of purchase | ☐ | ☐ | ☐ | ☐ | ☐ |
| Type of packaging | ☐ | ☐ | ☐ | ☐ | ☐ |
| Size of packaging | ☐ | ☐ | ☐ | ☐ | ☐ |
| Organic label | ☐ | ☐ | ☐ | ☐ | ☐ |
| Designation of origin | ☐ | ☐ | ☐ | ☐ | ☐ |

**CHARACTERISATION OF THE INTERVIEWEES**

16. Gender

☐ Woman ☐ Man

17. Age

| Between 18 and 24 | Between 25 and 34 | Between 35 and 49 | Between 50 and 64 | 65 or over |
|---|---|---|---|---|
| ☐ | ☐ | ☐ | ☐ | |

18. Members in your household: _____________

19. Level of studies

| Primary | Secondary | University (currently) | University (completed) |
|---|---|---|---|
| ☐ | ☐ | ☐ | ☐ |

20. Monthly FAMILY income (approx.)

| Less than €1,000 | €1,000-€1,999 | €2,000-€2,999 | €3,000-€3,999 | €4,000 or more |
|---|---|---|---|---|
| ☐ | ☐ | ☐ | ☐ | |
